# Commissioning and clinical positioning assessment of a novel surface‐guided radiation therapy (SGRT) system at a C‐Arm linear accelerator

**DOI:** 10.1002/acm2.70707

**Published:** 2026-08-06

**Authors:** Hui Khee Looe, Niklas Felix Hendrik Bartner, Björn Poppe, Kay C. Willborn

**Affiliations:** ^1^ University Clinic for Medical Radiation Physics Clinic for Radiation Therapy, Medical Campus Pius Hospital Carl von Ossietzky University Oldenburg Germany

**Keywords:** commissioning, positioning accuracy, SGRT, virtual lasers

## Abstract

**Background:**

Surface‐guided radiation therapy (SGRT) has been increasingly adopted in modern radiotherapy, enabling radiation‐dose‐free, marker‐free patient positioning with high accuracy. A novel SGRT system, LUNA 3D (LAP, Lüneburg, Germany), featuring a browser‐based interface, GPU‐accelerated surface reconstruction, high frame rates, large field‐of‐view and virtual laser projection capabilities, has been commissioned and implemented clinically.

**Purpose:**

This study reports comprehensive commissioning procedures and the associated results for the LUNA 3D system and evaluates its clinical impact on patient positioning accuracy for breast and pelvic cancer treatments.

**Methods:**

Commissioning tests were performed, including thermal drift, reproducibility, translational and rotational shift accuracy, camera occlusion assessment during gantry rotation, radiographic verification against cone‐beam computed tomography (CBCT), and End‐to‐End dosimetric testing. Test results were compared using two reference surfaces: one captured by LUNA 3D (SGRT‐reference) and the other derived from the CT external structure (SIM‐reference). A clinical evaluation was conducted to compare CBCT‐derived positioning corrections from 192 breast and 259 pelvic treatment datasets acquired in the periods before and after the clinical implementation of LUNA 3D, employing Welch's two‐sample t‐test and Cohen's *d* effect size.

**Results:**

The thermal drift within the operating temperature range is ≤0.4 mm for all three axes. Across the reproducibility and translational/rotational shift tests, maximum deviations were ≤0.3 mm translational and ≤0.2° rotational with the SGRT‐reference, and ≤0.8 mm translational and ≤0.2° rotational with the SIM‐reference, the latter attributable to the difference introduced by the CT‐derived reference surface. Radiographic verification showed agreement within 1.0 mm between LUNA 3D and CBCT corrections for both reference surfaces. End‐to‐End testing yielded CBCT residuals of 0.9–1.3 mm with 1.2% dosimetric deviation. All evaluated performance metrics are compared to the ESTRO‐ACROP and AAPM TG‐302 guidelines' tolerances. Clinical implementation resulted in significant positioning improvements: for breast treatments, 3D translational vector decreased 28.7% from 7.00 ± 4.35 mm to 4.99 ± 2.75 mm (*p* < 0.001, Cohen's *d* = 0.54); for pelvic treatments, 3D rotational vector decreased 24.0% from 2.31 ± 0.96° to 1.76 ± 0.67° (*p* < 0.001, Cohen's *d* = 0.66).

**Conclusions:**

LUNA 3D was successfully commissioned, meeting international SGRT guidelines. Clinical implementation produced statistically significant and clinically meaningful improvements in patient positioning accuracy with anatomical site‐specific benefits. These findings establish LUNA 3D as a reliable SGRT technology that enhances positioning accuracy in routine clinical practice.

## 1 INTRODUCTION

In recent years, surface‐guided radiation therapy (SGRT) has been increasingly adopted in modern radiotherapy practice, supporting patient setup alongside image‐guided radiation therapy (IGRT.^1^, ^2^ Surface‐guided systems utilize structured light projection and stereoscopic imaging technologies to reconstruct three‐dimensional patient surfaces in real‐time, enabling radiation‐dose‐free and marker‐free patient positioning guidance with high spatial accuracy.^3^ Beyond initial positioning, SGRT systems provide continuous real‐time monitoring capabilities and support advanced respiratory motion management techniques, such as deep‐inspiration breath‐hold (DIBH) for breast irradiation, where cardiac dose reduction can be achieved.^4^

The clinical advantages of SGRT have been demonstrated across multiple anatomical sites and treatment techniques.^5^, ^6^ Numerous studies have reported reductions in setup time following SGRT implementation compared to traditional laser‐based workflows.^4^ The improved reproducibility of patient positioning achieved through six‐degree‐of‐freedom (6DoF) offset measurements captured by SGRT systems can potentially reduce the frequency and necessity of ionizing radiation‐based image guidance.^3^, ^7^ The superior positioning accuracy enabled by SGRT could support tighter planning margins, improved target coverage, and enhanced normal tissue sparing.^4^, ^5^, ^7^ Studies comparing SGRT with conventional positioning methods have demonstrated that surface‐guided alignment yields comparable or enhanced accuracy to three‐point marker‐based localization, with performance varying by anatomical site.^7^

Recent international guidelines have established comprehensive recommendations for SGRT implementation. The AAPM Task Group 302 report provides detailed guidance on commissioning, clinical use, and quality assurance (QA) for SGRT systems, emphasizing the importance of risk assessment and systematic quality management programs.^8^ The ESTRO‐ACROP guideline complements these recommendations with European consensus on procurement, workflows, and site‐specific QA procedures.^1^ Both guidelines also emphasize that SGRT should be implemented as a complementary technology, but not as a substitute for image guidance, particularly for deep‐seated targets, where surface position may not reliably correlate with internal anatomy.

Recently, a novel SGRT system, LUNA 3D (LAP, Lüneburg, Germany), has been introduced for clinical use. The system features several technological advances including a browser‐based user interface enabling remote access and simplified workflow integration, GPU‐accelerated parallel computing for high‐speed surface reconstruction, large field‐of‐view coverage, high frame rates (>12 Hz) with latency below 250 ms, and a virtual laser concept that projects positioning guidance directly onto the recorded patient surface. These technical specifications position LUNA 3D as a competitive addition to existing SGRT systems, potentially addressing limitations of earlier‐generation systems.

In this work, comprehensive commissioning procedures for the LUNA 3D system installed at a C‐Arm linear accelerator are reported. The systematic testing approach encompasses thermal drift, reproducibility, translational and rotational shift accuracy, assessment of camera occlusion effects during gantry rotation, radiographic verification against cone‐beam computed tomography (CBCT), and End‐to‐End dosimetric testing. These commissioning activities establish baseline performance characteristics and validate the system's compliance with international guidelines. Furthermore, a clinical evaluation comparing positioning accuracy before and after the clinical implementation of LUNA 3D is presented, providing benchmark data for future investigations.

## 2 MATERIALS AND METHODS

### 2.1 SGRT System—LUNA 3D

The LUNA 3D SGRT system (LAP, Lüneburg, Germany) was installed at an Elekta C‐Arm linear accelerator (linac). The installation consists of three camera pods (two cameras per pod) utilizing blue structured‐light projection technology. The system employs multiple projectors that cast a defined pattern onto the patient's surface, which is captured by high‐resolution cameras from various angles. Through triangulation algorithms, a precise three‐dimensional surface model is reconstructed in real‐time. All measurements were performed using the software version 1.2.3.

**FIGURE 1:**
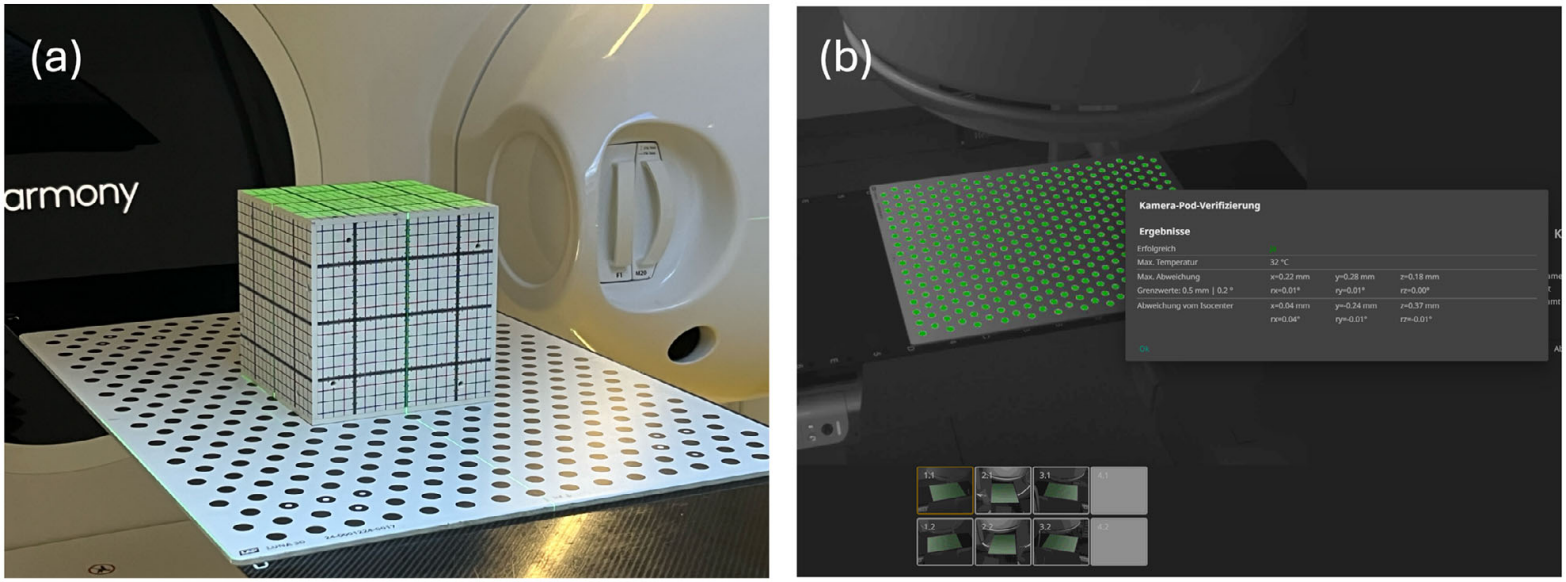
(a) Isocenter alignment setup for LUNA 3D system. Geometrical calibration plate with EASY CUBE phantom positioned at the linac isocenter. Both the geometrical plate and the phantom are aligned with the side‐wall (lateral) lasers, which set the cranio‐caudal position, and the sagittal (longitudinal) laser, which sets the left–right position; the vertical laser is aligned with the center of the phantom. (b) The daily quality assurance is performed using the geometrical plate alone. LUNA 3D software interface displaying the captured calibration plate position surface and the evaluated accuracy of the cameras’ calibrations, as well as the isocenter alignment.

The system's isocenter alignment is performed using a geometrical plate with an EASY CUBE phantom (LAP, Lüneburg, Germany) as shown in Figure 1a. Both the geometrical plate and the phantom are aligned with the side‐wall (lateral) lasers, which set the cranio‐caudal position, and the sagittal (longitudinal) laser, which sets the left–right position; the vertical laser is aligned with the center of the phantom. The daily QA is performed only with the geometrical plate as shown in Figure 1b. Thereby, the plate is aligned with the room lasers at a source‐to‐surface distance (SSD) of 100 cm. Using the images acquired with the six cameras, the calibration and the isocentric alignment accuracy are evaluated with an acceptance tolerance of 0.5 mm and 0.2°.

### 2.2 Test procedures

The tests conducted in this work include thermal drift, reproducibility, translational and rotational shift accuracy, impact of camera occlusion, radiographic verification, and End‐to‐End testing. The RUBY Modular Phantom (PTW Dosimetry, Freiburg, Germany) served as the primary phantom for commissioning measurements. This modular phantom features a geometric base design with well‐defined edges and surfaces suitable for surface tracking verification. Additionally, the phantom supports defined translational shifts as well as a combination of defined translational and rotational shifts, both with dedicated markings on the phantom's surface.

A CT scan of the phantom was performed using a goSim CT (Siemens Healthineers, Erlangen, Germany) with 1 mm slice thickness. The external structure of the phantom was generated using AI auto‐contouring in Elekta ONE Planning (Elekta AB, Stockholm, Sweden). For all tests, except the End‐to‐End testing (see Section 2.2.6), a treatment plan with the isocenter at the center of the phantom, as indicated by markings on the phantom's surface, was created.

#### 2.2.1 Thermal drift

To measure the temperature‐dependent drift of the LUNA 3D system, the calibration plate was placed at the isocenter position. Each of the three camera pods continuously and independently measured the position of the calibration plate. During the measurements, the projectors were turned on and off consecutively for 60 min to introduce and remove thermal load. The procedure was repeated twice. The 3D positions, along with the temperatures recorded by the internal built‐in sensors, were registered.

#### 2.2.2 Reproducibility

**FIGURE 2:**
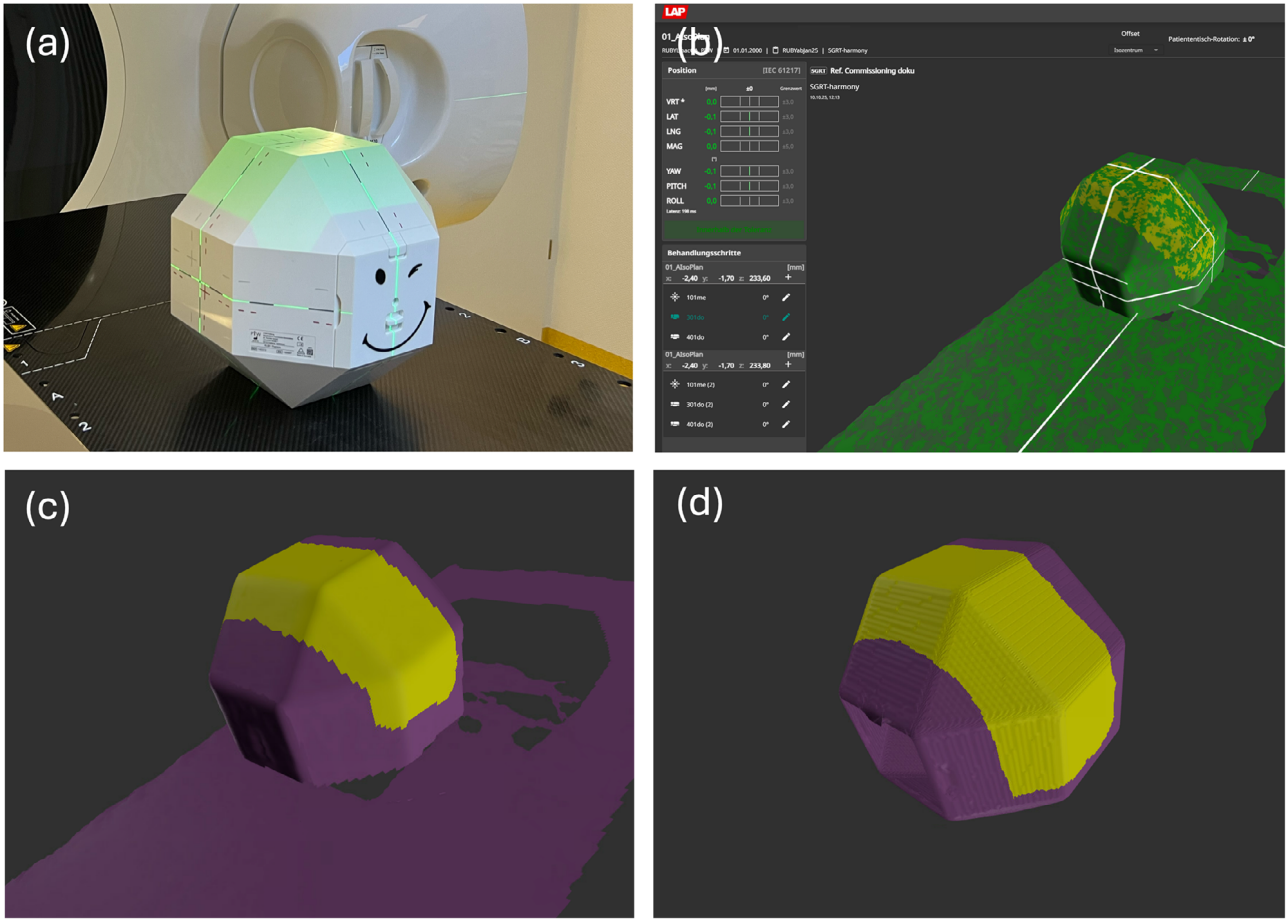
Reproducibility test setup with RUBY phantom. (a) The phantom is positioned at the isocenter using room laser alignment with surface markings. (b) LUNA 3D software interface screenshot showing the live surface (green) and measured 6DoF values. (c) SGRT‐reference: reference surface captured by LUNA 3D. (d) SIM‐reference: reference surface derived from the external structure of the phantom's CT scan. The ROI defined for tracking (derivation of 6DoF values) is indicated as a yellow surface overlay on the reference surface.

The reproducibility measurements were performed by first positioning the RUBY phantom at the isocenter, aligning the surface markings on the phantom with the room lasers (Figure 2a). A reference surface was then captured with LUNA 3D (SGRT‐reference). Subsequently, the phantom was removed from the couch and repositioned five times. The 6DoF positioning deviations from the reference were recorded, and the maximum deviation across all repetitions was documented for each DoF (Figure 2b).

The same test was repeated using the external structure derived from the phantom's CT scan as the reference surface (SIM‐reference). Similarly, the phantom was repositioned five times. The SGRT‐reference and the SIM‐reference are both shown in Figure 2c,d, respectively, where the region of interest (ROI) is shown as a yellow surface.

During the reproducibility test, the effect of room brightness was initially evaluated. The 6DoF values remained consistent within 0.1 mm and 0.1° when switching between the dimmest and brightest lighting conditions. These small fluctuations correspond to the system's natural temporal dynamics. Consequently, all later tests were conducted under the standard ambient lighting setup used in clinical practice.

#### 2.2.3 Translational and rotational shifts

**FIGURE 3:**
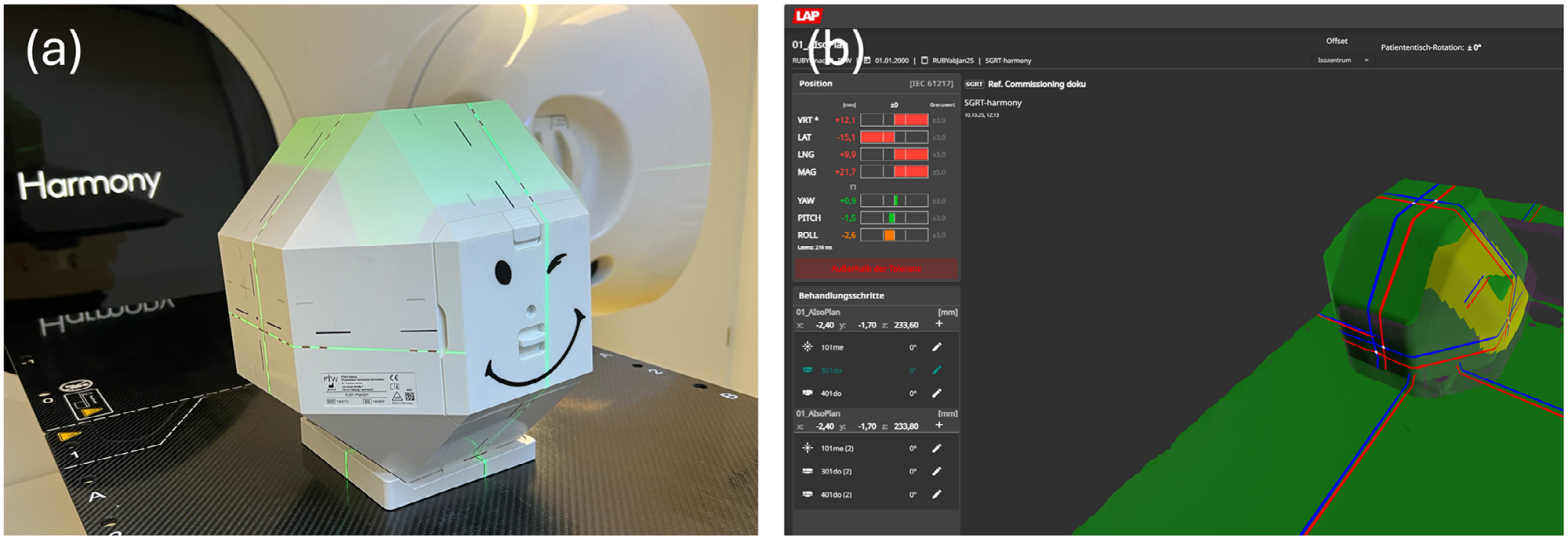
Translational and rotational shift accuracy testing setup. (a) RUBY phantom positioned on a dedicated tilting base providing predefined translational shifts (12 mm vertical, 10 mm longitudinal, 15 mm lateral) and rotational shifts (1° yaw, –1.5° pitch, –2.5° roll) relative to the isocenter position. (b) Cropped LUNA 3D software interface displaying the six 6DoF positioning deviations from the reference surface. The deviations between the fixed red virtual lasers indicating the room isocenters and the blue virtual lasers indicating the isocenter defined in the phantom (treatment plan) also reflect these introduced shifts. In clinical practice, these serve as intuitive visual feedback to the user, whose aim is to align the blue lines with the red lines.

Using the same reference surfaces as in Section 2.2.2, the RUBY phantom was repositioned by placing it on the dedicated tilting base, with a different set of markers on the phantom surface that shift it by predefined translations and rotations relative to the isocenter position specified by the manufacturer. The setup is shown in Figure 3a. The procedure was repeated five times. The 6DoF values were recorded (Figure 3b), where the maximum deviation from the expected values across all repetitions was documented for each DoF.

An additional rotational accuracy test to assess the LUNA 3D system's ability to quantify treatment couch rotation was performed using the EASY CUBE phantom, combined with a dedicated rotational base that allows the phantom to be isocentrically rotated at nine different angular positions, from –90° to 90° in 22.5° increments. For each position, the angle was entered into the LUNA 3D software as couch rotation, and the remaining yaw rotation value reported by LUNA 3D was recorded.

#### 2.2.4 Impact of camera occlusion

Using the same setup and reference as in Section 2.2.2, the maximum deviation of the measured 6DoF values during gantry rotation from the baseline values at 0° with retracted and extended kV X‐ray source as well as the kV and MV electronic portal imaging devices (EPIDs) was recorded, alongside the gantry angle when this occurred.

#### 2.2.5 Radiographic verification

For the radiographic verification, the RUBY phantom was deliberately positioned with defined translational shifts. The same reference surfaces as in Section 2.2.2 were used. A CBCT was acquired with the phantom in the shifted position. In the ideal case, the LUNA 3D corrections should be identical to the CBCT‐based corrections. The differences between the LUNA 3D‐and CBCT‐derived positioning corrections were computed. Subsequently, the treatment couch was shifted according to CBCT‐derived offsets, with residual positioning errors documented to account for the mechanical precision of the couch positioning system.

#### 2.2.6 End‐to‐end dosimetric testing

**FIGURE 4:**
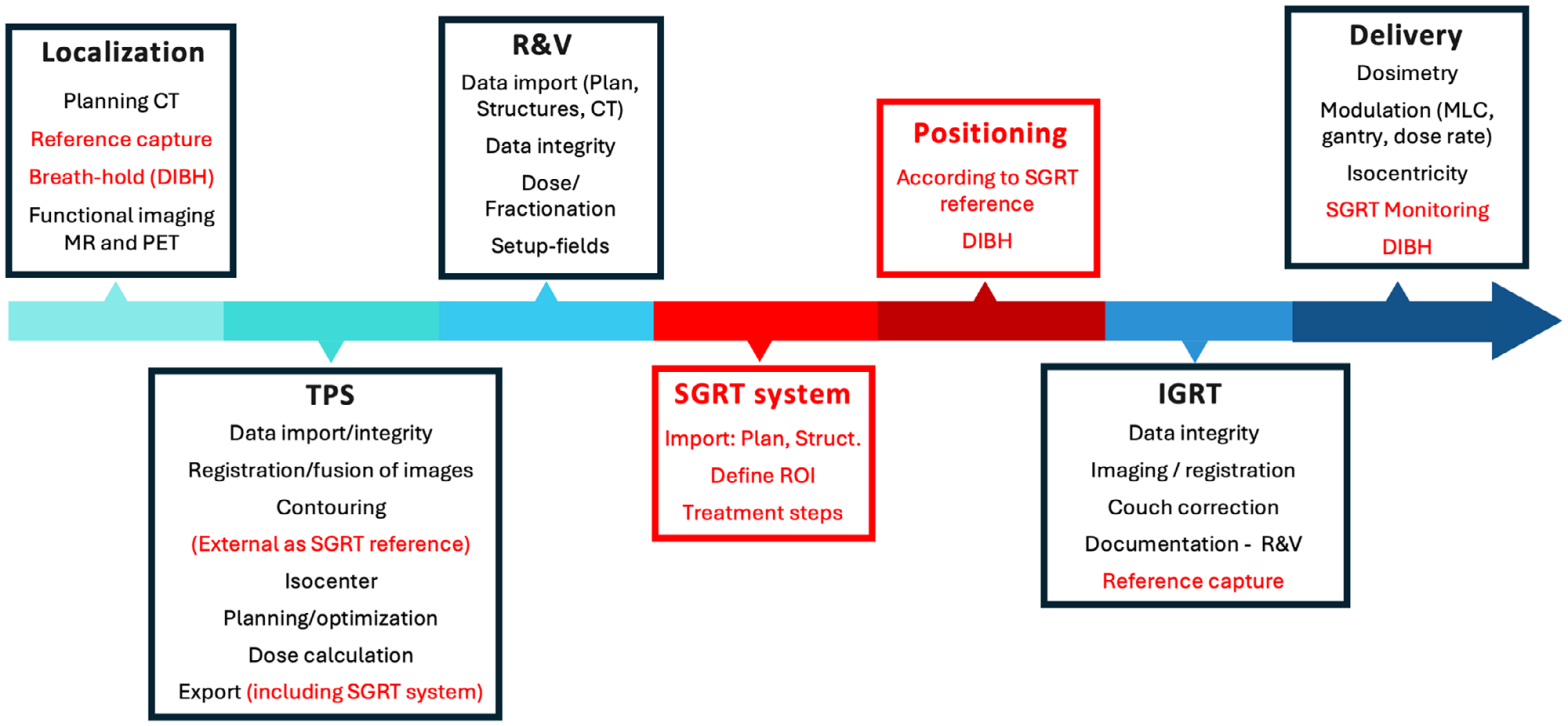
End‐to‐End dosimetric testing workflow incorporating laser‐free positioning with LUNA 3D. Schematic representation of the complete workflow from CT simulation through treatment delivery and dosimetric verification. Steps highlighted in red indicate LUNA 3D‐specific procedures. The workflow validates the complete laser‐free positioning capability from simulation through treatment.

The procedure for the End‐to‐End dosimetric testing is depicted in Figure 4. The procedure was derived based on an existing workflow using the RUBY phantom in combination with the SystemQA insert.^9^, ^10^ The test has been adapted to incorporate the laser‐free positioning workflow with LUNA 3D (steps in red).

The CT scan of the RUBY phantom with the SystemQA insert was performed as described previously, where the external structure of the phantom was generated using AI auto‐contouring as in clinical routine. Subsequently, the CT and structure set were imported into Monaco (version 6.2.3) treatment planning system (TPS) (Elekta AB, Stockholm, Sweden). A treatment plan was created with the isocenter placed at an arbitrary position within the phantom. This ensures that the phantom at the linac cannot be positioned with the help of external markings on the phantom.

The resulted RT‐Plan and RT‐Structure set were exported to the LUNA 3D server to create a corresponding treatment course with a reference surface derived from the external structure (SIM‐reference). The dataset was also exported to the Mosaiq (version 3.1.1.0) Record and Verify (R&V) system (Elekta AB, Stockholm, Sweden) as well as the linac X‐ray volume imaging (XVI) system (Elekta AB, Stockholm, Sweden) for CBCT acquisition. All other preparation steps were carried out as in clinical routine.

At the linac, the phantom was positioned solely using LUNA 3D with the imported SIM‐reference. During the whole process, the room lasers were switched off. Subsequently, a CBCT was acquired to verify the phantom position. Finally, the plan was irradiated, and the dose was measured using a cross‐calibrated microDiamond detector (PTW Dosimetry, Freiburg, Germany) placed in the SystemQA insert at the center of the target, which was then compared to the calculated dose.

### 2.3 Evaluation of clinical performance

Five months after the clinical implementation of the LUNA 3D system, a retrospective analysis was performed by comparing the CBCT‐derived positioning corrections of two patient cohorts: pre‐LUNA 3D implementation and post‐LUNA 3D implementation. The CBCT‐based translational shifts (lateral, longitudinal, and vertical) and rotational shifts (pitch, roll, and yaw) were converted to absolute values for analysis.

To provide a comprehensive assessment of overall positioning accuracy, 3D vector magnitudes were calculated for both translational and rotational shifts using the Euclidean distance formula: 3D vector = $\sqrt{a^{2}+b^{2}+c^{2}}$, where *a*, *b*, and *c* represent the three orthogonal components (lateral, longitudinal, and vertical for translation; pitch, roll, and yaw for rotation). This metric captures the combined positioning error across all dimensions and provides a clinically relevant measure of overall geometric uncertainty.

Sample sizes for the retrospective analysis comprised 106 CBCT datasets without LUNA and 86 datasets with LUNA for breast treatments, and 143 CBCT datasets without LUNA and 116 datasets with LUNA for pelvic treatments. Breast treatments include whole breast, partial breast, and chest wall irradiations under free breathing, whereas pelvic treatments include prostate, rectum, and cervical irradiations. CBCTs were usually performed 2–3 times per week. Within each patient group, the same immobilization and imaging protocol was used.

Descriptive statistics including mean and standard deviation were calculated for each directional component and 3D vector magnitude. Statistical comparisons between the positioning data with and without LUNA 3D were performed using Welch's two‐sample *t*‐test with a significance level of *α* = 0.05. Welch's *t*‐test was selected as it does not assume equal variances between groups and is robust for comparing independent samples with potentially different population variances. The magnitudes of the translational and rotational vectors were pre‐specified as distinct primary endpoints for each site and analyzed separately; accordingly, no correction for multiple comparisons was applied. Effect sizes were quantified using Cohen's *d* to assess the practical significance of observed differences, with interpretation following standard conventions: small effect (*d* = 0.2), medium effect (*d* = 0.5), and large effect (*d* = 0.8).

## 3 RESULTS

### 3.1 Thermal drift

**FIGURE 5:**
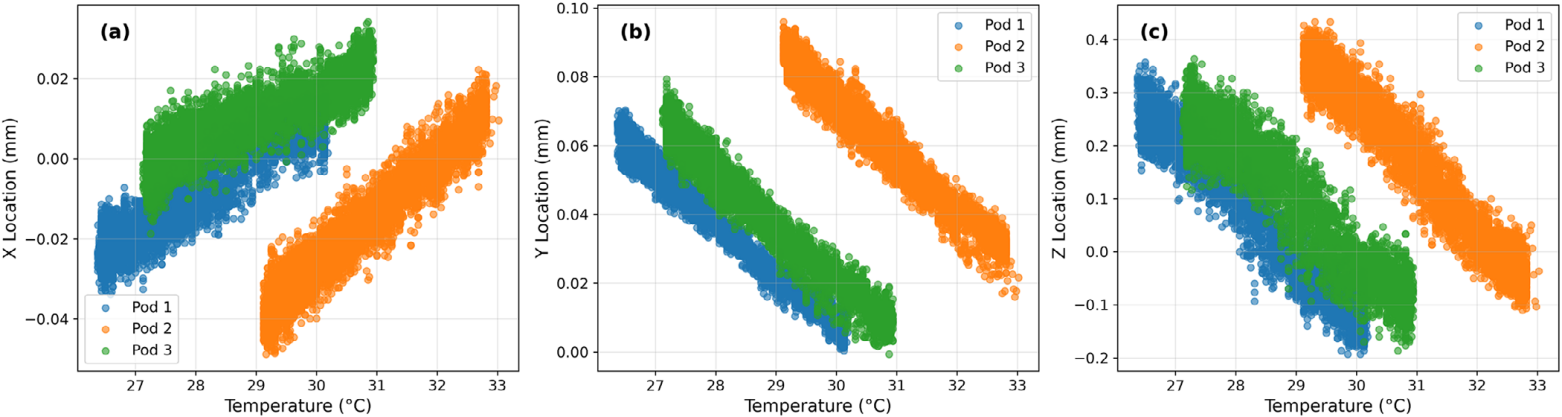
Spatial location drift in (a) X‐, (b) Y‐, and (c) Z‐direction measured by each camera pod (Pod 1–3) as a function of the corresponding temperature registered by the built‐in sensor in the camera pod. All location values are normalized to the first measurement point.

Figure 5 presents the measured X, Y, and Z positions of the calibration plate by each camera pod as scatter plots against the registered temperature of the camera pod. To better assess the effect of thermal drift, all position values were normalized to the first measured values. The thermal drift effects introduced by the projectors occur mainly in the Z direction (distance to camera pod) with a maximum magnitude of 0.4 mm, while the effects are minimal (≤0.1 mm) in the X and Y directions.

### 3.2 Reproducibility

The LUNA 3D system demonstrated high reproducibility across repeated positioning attempts. When utilizing SGRT‐reference, the maximum deviations remained within 0.1 mm for the vertical direction, 0.2 mm for longitudinal and lateral directions, and 0.1° for all rotational dimensions across five independent measurements.

When using the SIM‐reference, the system showed larger maximum deviations reaching –0.8 mm vertical, 0.2 mm longitudinal, and 0.4 mm lateral for translational dimensions. Rotational deviations remained within 0.2° for all three rotational DoF across five independent measurements. Notably, SIM‐reference results revealed a systematic vertical offset of –0.7 mm, while the longitudinal and lateral directions showed smaller biases of +0.2 and +0.3 mm, respectively.

### 3.3 Translational and rotational shifts

Using the dedicated tilting base, the RUBY phantom was shifted with known displacements of 12 mm vertical, 10 mm longitudinal, and 15 mm lateral, combined with rotations of 1° yaw, –1.5° pitch, and –2.5° roll. Using SGRT‐reference, detection accuracy for predefined translational and rotational shifts demonstrated good agreement with expected values, with maximum deviations of –0.2 mm vertical, 0.1 mm longitudinal, –0.3 mm lateral, as well as –0.2° yaw, 0° pitch, and –0.1° roll.

When using the SIM‐reference, the system demonstrated larger translational deviations. Maximum deviations from expected values reached –0.8 mm vertical, 0.3 mm longitudinal, and –0.5 mm lateral across five repetitions. The larger deviations, especially in the vertical direction, are consistent with the differences associated with SIM‐reference as reported in Section 3.2. Rotational accuracy remained high with maximum deviations of –0.2° yaw, 0° pitch, and ‐0.1° roll.

In the treatment couch rotational accuracy test, the LUNA 3D recorded yaw rotation deviations were smaller than 0.2° for all tested rotations. The translational offsets remained within 0.1 mm for all the phantom's positions.

### 3.4 Impact of camera occlusion

Using SGRT‐reference with all components retracted, maximum deviations remained within 0.1 mm for all translational directions and 0.1° for all rotational dimensions across the full 360° gantry rotation. With components fully extended (maximum occlusion), identical performance was observed with no detectable differences in measurement accuracy.

Using SIM‐reference and components retracted, maximum deviations reached –0.9 mm vertical (at –15°), –0.6 mm longitudinal (at –85°), and 1.0 mm lateral (at –19°) for translational dimensions. Rotational deviations were all within 0.2°. With imaging‐components fully extended, maximum translational deviations increased slightly to –1.0 mm vertical (at –160°), 1.0 mm longitudinal (at –140°), and –1.2 mm lateral (at –161°). Rotational deviations increased slightly to 0.3°.

### 3.5 Radiographic verification

Table 1 shows the comparison of LUNA 3D and CBCT‐based corrections for using both SGRT‐reference and SIM‐reference. The RUBY phantom was positioned with known translational shifts of –18 mm vertical, –14 mm longitudinal, and 25 mm lateral.

**TABLE 1:** Radiographic verification comparing LUNA 3D and CBCT‐based positioning corrections. The RUBY phantom was positioned with predefined shifts of –18.0 mm vertical, –14.0 mm longitudinal, and 25.0 mm lateral. Results are shown for tests (a) using SGRT‐reference and (b) using SIM‐reference across three repetitions. LUNA 3D and CBCT corrections are shown before couch correction, with remaining LUNA 3D deviations and couch residuals documented after couch correction. Maximum deviations represent the largest discrepancies across all repetitions. All values in mm.

(a) SGRT‐reference								
Before couch correction					After couch correction			
Repetition		Vert	Long	Lat		Vert	Long	Lat
1	LUNA 3D	−18.1	−13.9	24.7	LUNA 3D	0.1	0.2	−0.9
	CBCT	−18.6	−13.6	25.5	Couch residue	−0.2	0.3	0.1
2	LUNA 3D	−18.2	−13.9	24.8	LUNA 3D	0.4	0.1	−0.9
	CBCT	−18.9	−14.1	25.7	Couch residue	0.2	−0.2	0.0
3	LUNA 3D	−17.9	−14.2	24.6	LUNA 3D	0.5	−0.7	−1.5
	CBCT	−18.4	−13.7	25.6	Couch residue	−0.1	−0.3	−0.2
	Max dev.	0.7	−0.5	−1.0	Max dev.	0.6	−0.4	−1.3

(b) SIM‐reference								
Before couch correction					After couch correction			
Repetition		Vert	Long	Lat		Vert	Long	Lat
1	LUNA 3D	−18.5	−13.9	25.4	LUNA 3D	−0.4	0.3	−0.8
	CBCT	−18.4	−13.7	25.6	Couch residue	0.0	0.3	−0.1
2	LUNA 3D	−18.6	−13.6	25.2	LUNA 3D	−0.6	0.4	−0.8
	CBCT	−18.4	−13.7	25.7	Couch residue	−0.1	0.1	−0.3
3	LUNA 3D	−18.6	−13.5	24.9	LUNA 3D	−0.6	−0.6	−0.6
	CBCT	−18.3	−13.2	25.3	Couch residue	0.0	−0.4	−0.1
	Max dev.	−0.3	−0.3	−0.5	Max dev.	−0.6	0.3	−0.7

With SGRT‐reference, the maximum deviations between the corrections registered by LUNA 3D and by CBCT are 0.7 mm vertical, –0.5 mm longitudinal, and –1 mm lateral. All rotation values agree within 0.3°. Compared with the test reported in Section 3.3, the results are subject to kV and laser isocenter congruence, CT‐CBCT registration, and isocenter placement in the treatment plan. After the couch correction, the maximum couch residue as indicated on the linac console was ±0.3 mm. The maximum differences between the remaining LUNA 3D corrections and couch residue values after couch correction amount to 0.6 mm vertical, –0.4 mm longitudinal, and –1.3 mm lateral. This larger lateral value combines the LUNA 3D‐CBCT discrepancy (–1.0 mm) with possible couch‐positioning imprecision.

Using the SIM‐reference, the maximum deviations between LUNA 3D‐derived corrections and CBCT corrections were –0.3 mm vertical, –0.3 mm longitudinal, and –0.5 mm lateral. All rotation values agreed within 0.3°. Following couch correction, the remaining LUNA 3D‐indicated deviations and the couch residuals resulted in a maximum total difference of –0.6 mm vertical, 0.3 mm longitudinal, and –0.7 mm lateral. Notably, these differences using SIM‐reference are smaller than those observed with SGRT‐reference, despite the reported vertical bias, suggesting a complex interplay between the additional uncertainty components mentioned earlier and the reference surface used.

### 3.6 End‐to‐end dosimetric testing

**FIGURE 6:**
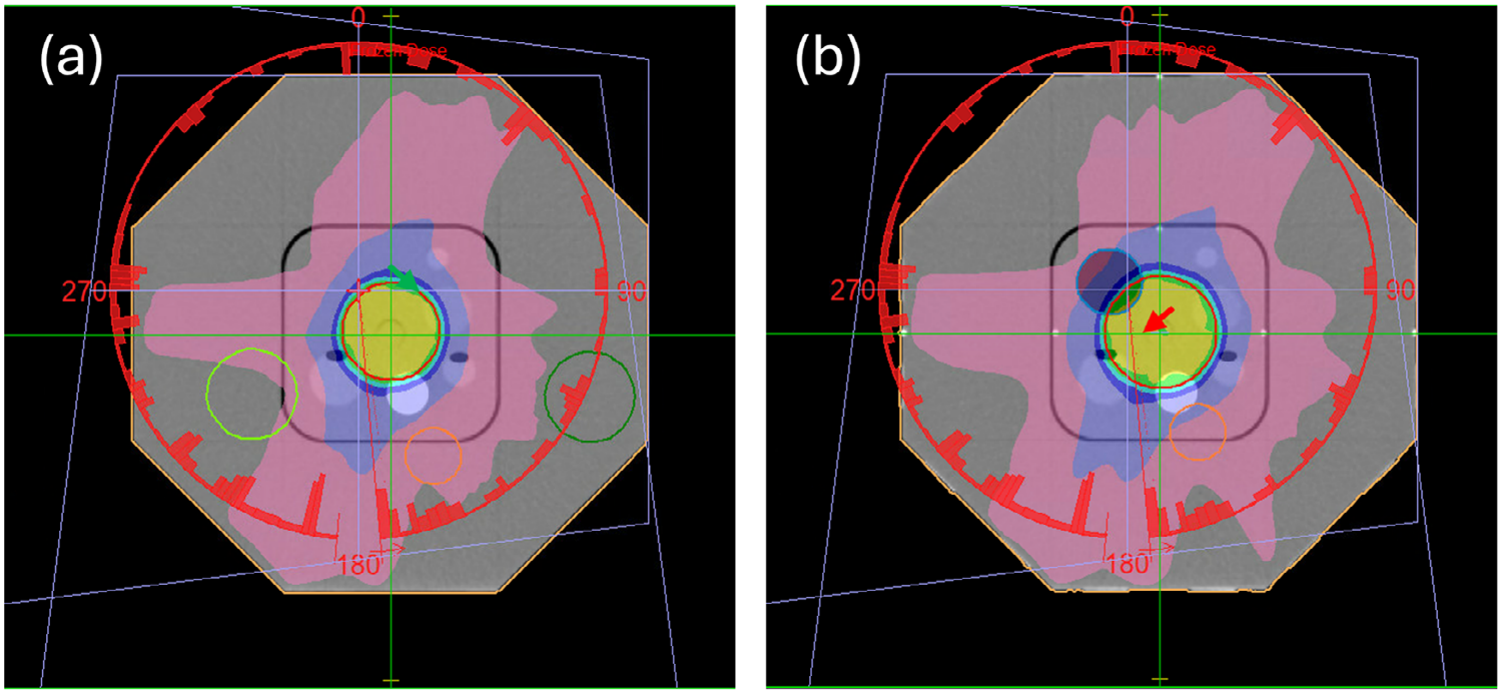
End‐to‐End testing dosimetric verification. (a) Transversal CT slice of the RUBY phantom showing the treatment isocenter (green arrow) positioned at an arbitrary location within the phantom to prevent external marker‐based positioning. (b) Target volume centered at the measurement position, where the microDiamond detector is placed in the SystemQA insert at the center of the phantom, indicated by the red arrow.

Figure 6a shows the transversal slice of the RUBY phantom, in which the isocenter has been defined as indicated by the green arrow. The dose was measured at the center of the phantom, at which the target was also centered, as indicated by the red arrow in Figure 6b. The CBCT‐based corrections after the phantom was positioned solely with LUNA 3D amounted to 0.9 mm vertical, –0.6 mm longitudinal and –1.3 mm lateral. The rotation values agree within 0.5° (yaw 0°, pitch 0.1°, roll 0.5°). The values are comparable to the results of radiographic verification presented in the previous Section 3.5 using SIM‐reference. The measured dose using the microDiamond detector (3.106 Gy) agreed with the calculated dose (3.142 Gy) with a deviation of –1.2%.

### 3.7 Evaluation of clinical performance

**FIGURE 7:**
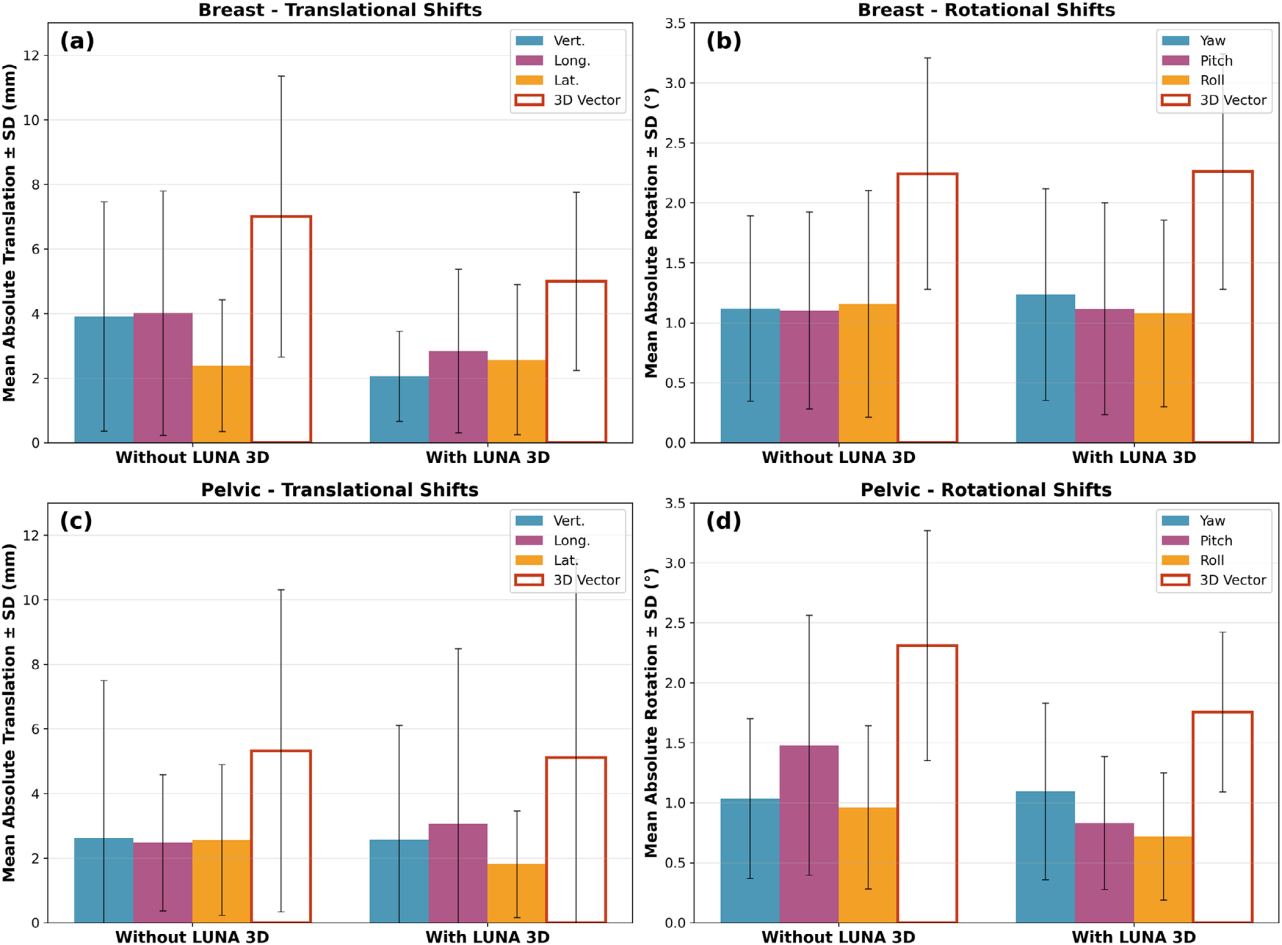
Positioning accuracy with and without LUNA 3D surface guidance. Bar plots comparing mean absolute positioning errors (±SD) for (a, b) breast and (c, d) pelvic treatments. (a, c) Translational shifts in millimeters and (b, d) rotational shifts in degrees are shown separately. The individual directional components are shown in filled color bars, while the 3D vector magnitudes (calculated as Euclidean distance: $\sqrt{a^{2}+b^{2}+c^{2}})$, are shown as hollow bars. For breast treatments, LUNA 3D significantly reduced translational 3D vector errors by 28.7% (7.00 ± 4.35 mm vs. 4.99 ± 2.75 mm, *p* < 0.001, Cohen's *d* = 0.54) but had no significant effect on rotational errors (*p* = 0.897). For pelvic, LUNA significantly reduced rotational 3D vector errors by 24.0% (2.31 ± 0.96° vs. 1.76 ± 0.67°, *p* < 0.001, Cohen's *d* = 0.66) but had no significant effect on translational errors (*p* = 0.771).

Retrospective analysis of CBCT positioning corrections revealed measurable improvements in positioning accuracy following clinical implementation of LUNA 3D for both breast and pelvic cancer treatments, as presented in Figure 7.

For breast cancer patients, significant improvements were observed in overall translational positioning accuracy. The 3D translational vector decreased significantly from 7.00 ± 4.35 mm to 4.99 ± 2.75 mm, representing a 28.7% improvement (*t* = 3.89, *p* < 0.001, Cohen's *d* = 0.54). This medium effect size indicates a clinically meaningful improvement in translational positioning accuracy. Individual directional components showed vertical deviations decreasing from 3.91 ± 3.55 mm to 2.06 ± 1.39 mm (47.3% reduction), longitudinal deviations reducing from 4.01 ± 3.78 mm to 2.84 ± 2.52 mm (29.2% reduction), and lateral deviations increasing slightly from 2.39 ± 2.04 mm to 2.57 ± 2.32 mm. Rotational positioning for breast treatments remained stable, with the 3D rotational vector showing no significant change from 2.24 ± 0.96° to 2.26 ± 0.98° (*t* = –0.13, *p* = 0.897, Cohen's *d* = –0.02). Individual rotational components demonstrated yaw increasing from 1.12 ± 0.77° to 1.23 ± 0.88°, pitch increasing marginally from 1.10 ± 0.82° to 1.12 ± 0.88°, and roll decreasing from 1.16 ± 0.94° to 1.08 ± 0.78°.

For pelvic cancer patients, LUNA 3D implementation also resulted in significant improvements in rotational positioning accuracy. The 3D rotational vector decreased from 2.31 ± 0.96° to 1.76 ± 0.67°, representing a 24.0% improvement (*t* = 5.47, *p* < 0.001, Cohen's *d* = 0.66). This medium effect size indicates a clinically meaningful improvement in rotational positioning accuracy. Individual rotational components showed pitch decreasing substantially from 1.48 ± 1.08° to 0.83 ± 0.56° (43.8% reduction), roll reducing from 0.96 ± 0.68° to 0.72 ± 0.53° (25.2% reduction), and yaw increasing slightly from 1.03 ± 0.67° to 1.09 ± 0.74°. Translational positioning for pelvic treatments showed minimal change, with the 3D translational vector decreasing non‐significantly from 5.32 ± 4.98 mm to 5.11 ± 6.14 mm (*t* = 0.29, *p* = 0.771, Cohen's *d* = 0.04). Individual directional components demonstrated lateral positioning decreasing from 2.56 ± 2.33 mm to 1.81 ± 1.65 mm (29.3% reduction), vertical positioning reducing slightly from 2.62 ± 4.88 mm to 2.57 ± 3.54 mm, and longitudinal positioning increasing from 2.48 ± 2.11 mm to 3.05 ± 5.43 mm.

These findings demonstrate that the LUNA 3D implementation enhanced initial patient positioning accuracy in routine clinical workflows, with statistically significant and clinically meaningful improvements, particularly for breast translational and pelvic rotational positioning.

## 4 DISCUSSION

### 4.1 Technical performance

The thermal drift effects introduced by the projectors occur predominantly in the Z direction (distance to camera pod) with a maximum magnitude of 0.4 mm, while remaining minimal (≤0.1 mm) in the X and Y directions. The LUNA 3D system achieved high reproducibility testing with SGRT‐reference, with maximum deviations of 0.1 mm vertically, 0.2 mm in longitudinal and lateral directions, and 0.1° in all rotational dimensions. When using SIM‐reference, the larger deviations in the translational shifts of 0.8 mm vertical, 0.2 mm longitudinal, 0.4 mm lateral might be attributable to additional influences related to the differences in the surfaces of different origins (SGRT and CT), as well as pixel discretization in CT and its effect on the surface delineations in CT. However, the maximum rotational deviation remained small at 0.2°. The translational and rotational shift accuracy tests showed a maximum deviation of 0.3 mm translational and 0.2° rotational. When SIM‐reference was used, the maximum translational deviations reached –0.8 mm, while rotational accuracy remained the same at –0.2°. Independent treatment couch rotation test resulted in agreement better than 0.2° (yaw).

The impact of camera occlusion during gantry rotation was minimal with SGRT‐reference, with deviations remaining at or below 0.1 mm and 0.1° throughout the full 360° rotation arc with imaging components retracted or extended. In contrast, the use of SIM‐reference showed larger gantry angle‐dependent influences, with maximum translational deviations of 1.2 mm occurring at specific angles for a short duration (1–2 s).

The radiographic verification tests comparing LUNA 3D‐derived corrections and CBCT‐based corrections resulted in maximum differences of 0.7 mm vertically, –0.5 mm longitudinally, and –1.0 mm laterally before couch correction. These discrepancies are within expected ranges when considering the cumulative uncertainties from congruence of the kV‐imaging and laser isocenters as well as CT‐CBCT registration algorithms. After the couch corrections were applied, residual couch positioning errors of up to 0.3 mm were observed, accounting for mechanical couch positioning precision. Radiographic verification tests using SIM‐reference reflected slightly better results, where the maximum deviations are all within 1 mm as recommended.^1^

The End‐to‐End testing yielded CBCT‐derived residual positioning errors of 0.9 mm vertically, 0.6 mm longitudinally, and 1.3 mm laterally. The measured dose deviated from the TPS‐calculated dose by only 1.2%. This lies within the ±2% tolerance commonly applied to End‐to‐End testing^11^ and demonstrates that surface‐guided positioning, without traditional laser alignment, can achieve clinically acceptable accuracy for radiation delivery.

Liu et al.^12^ used a LUNA 3D three‐camera pod setup, combined with a 6DoF robot and infrared‐based phantom verification, to report translational noise RMSE values of 0.04–0.06 mm and rotational noise RMSE values of 0.04°, with a maximum difference of 0.14 mm compared to infrared tracking across various surface colors, ambient lighting conditions, and ROI sizes. This was achieved at approximately 12.5 fps. Meanwhile, Lew et al.^13^ installed a LUNA 3D system on a full‐rotation proton gantry with a linear camera arrangement, observing a moving‐average drift of 0.0233 mm over 200 min. Their results showed static and dynamic localization deviations mostly below 0.2 mm, with a maximum dynamic deviation of 0.734 mm, End‐to‐End agreement within 0.8 mm, and gantry‐rotation stability within 0.2 mm. Both studies demonstrated comparable system performance that meets clinical standards.

The commissioning test results shown in this work are summarized in Table 2, following the ESTRO‐ACROP guidelines (Table 3).^1^ A comparison with the recommendations in AAPM TG‐302 (Table 4)^8^ is also included.

**TABLE 2:** Summary of LUNA 3D commissioning test results compared against ESTRO‐ACROP and AAPM TG‐147/TG‐302 recommendations.

Parameter	Label	Test	ESTRO‐ACROP tolerance	This work	Comparison with AAPM TG‐147/TG‐302
Static accuracy	A1	Isocenter coincidence (SGRT & kV‐imaging)	1 mm/1°; SRS: 0.5 mm/0.5°	1 mm/0.3°	Imaging isocenter coincidence ≤2 mm; ≤1 mm for SRS/SBRT
	A2	Translational shifts	1 mm	0.8 mm	Localization accuracy over a reasonable clinical range (∼100 mm): 2 mm; 1 mm for SRS/SBRT
	A3	Rotational shifts	1°	0.2°	No explicit numeric tolerance
	A4	Impact of camera occlusion	1 mm/1°	1.2 mm^a^/0.3°	Camera system characteristics, no explicit numeric tolerance
	A5	Couch rotation	1 mm/0.5°	0.1 mm/0.2°	No explicit numeric tolerance
	A6	Setup/loading position	1 mm/1°	NA^b^	No explicit numeric tolerance
Dynamic accuracy	D1‐D5	Beam‐hold, tracking, respiratory trace, trigger and frame‐rate performance		Not assessed^c^	
End‐to‐End Positioning	E1	End‐to‐End positioning	2 mm/1°	1.3 mm/0.5° 1.2% dose agreement^d^	Dose change ≤1%; ≤2% including beam hold, Winston–Lutz including SGRT for SRS < 1 mm
System performance	P1	Thermal drift	0.5 mm, 0.5°	≤0.4 mm/NA	Warm‐up: localization stability within 2 mm over 1 h; once stable, reproducibility within 1 mm
	P2	Room‐light impact	1 mm/1°	0.1 mm/0.1°	Camera system characteristics, no explicit numeric tolerance
	P3	Field‐of‐view characterization	Pass	Pass^e^	Camera system characteristics, measure localization FOV, meet vendor specifications
	P4	Surface image quality	Pass	Pass^e^	Camera system characteristics
	P5	System integration with peripherals	Pass	Pass^e^, ^f^	Interface with peripheral systems, couch shift functionality
	P6	Patient interface (coaching)	Pass	Pass^e^	Interface with peripheral systems
	P7	Registration‐matrix quality	Pass	Pass^e^	Camera system characteristics
Safety	S1	Interlocks & beam‐inhibit	Pass	NA^c^	Interface with peripheral systems, beam delivery functionality
	S2	Data import & export	Pass	Pass^e^	Interface with peripheral systems, integrity of data transferred, confirm isocenter coordinate transfers
	S3	Database, backup & security	Pass	Pass^e^	Interface with peripheral systems
	S4	User configuration & roles	Pass	Pass^e^	
	S5	Mechanical integrity	Pass	Pass^e^	
Documentation	R1	Export patient or QA reports	Pass	Pass^e^	
	R2	User manuals	Pass	Pass^e^	Complemented by SOPs should include training, indications, and be updated as experience grows

^a^ The maximum deviation of 1.2 mm is slightly higher than the tolerance, observed with SIM‐reference and fully extended imaging components.

^b^ Loading position not implemented for clinical workflow.

^c^ Not applicable to the current software version (1.2.3) with limited interoperability; dynamic/gated delivery not yet supported (see Section 4.3).

^d^ The 1.2% is the absolute agreement between the microDiamond‐measured and TPS‐calculated dose for the complete SGRT‐positioned workflow.

^e^ These aspects are not performed explicitly, but are prerequisites for other tests, especially for completing the End‐to‐End testing.

^f^ Not fully implemented due to limited interoperability with R&V system.

Both the ESTRO‐ACROP and AAPM TG‐302 recommend that routine QA tests, listed separately, be conducted on a daily, weekly, monthly, or yearly schedule. These tests are similar to those shown in Table 2 and are essential for verifying the SGRT system's performance post‐commissioning. Additionally, it is important to note that these routine tests should be complemented by tests specific to each vendor.

### 4.2 Clinical accuracy

Statistical analysis identified two significant improvements (both *p* < 0.001 with medium effect sizes): breast translational positioning (28.7% reduction in 3D vector magnitude) and pelvic rotational positioning (24.0% reduction in 3D vector magnitude).

For breast cancer treatments, improvement was driven primarily by enhanced vertical and longitudinal positioning. Rotational positioning for breast treatments remained stable, with the 3D rotational vector showing no significant change. For pelvic cancer treatments, the rotational improvement was driven primarily by substantial reductions in pitch (43.8% from 1.48° to 0.83°) and roll (25.2% from 0.96° to 0.72°) rotations.

Recent literature supports these findings. Jeong et al. analyzed 184 breast cancer patients comparing marker‐less SGRT with traditional skin marks, reporting mean 3D vector errors of 5.2 mm with SGRT.^14^ Svestad et al. conducted a randomized crossover trial of 25 breast cancer patients, reporting 3D vector errors of 5.1 mm with SGRT versus 5.3 mm with skin marks, demonstrating statistical equivalence.^15^ Rigley et al. evaluated tattoo‐free SGRT positioning in 43 breast patients, demonstrating significantly improved accuracy compared to conventional methods (4.7 mm vs. 5.2 mm, *p* = 0.04), with more pronounced benefits for DIBH treatments (4.5 mm vs. 7.6 mm, *p* < 0.001).^16^ Kang et al. reported mixed directional performance with SGRT showing improved vertical accuracy, but larger longitudinal errors compared to skin marks.^17^ The clinical performance of LUNA 3D aligns with these recent comparative evaluations of SGRT systems, where the present study extends beyond breast‐focused literature by evaluating pelvic treatments, revealing differential effectiveness across anatomical sites and positioning dimensions.

The current results also highlight that SGRT should be implemented as a complement to, rather than a complete replacement for, imaging‐based position verification. For treatment sites where internal target position may not correlate reliably with external surface anatomy or lung tumors subject to organ motion, combined SGRT and CBCT workflows remain the standard of care. The LUNA 3D system's ability to reduce but not eliminate the need for imaging verification aligns with current clinical guidelines recommending hybrid positioning strategies that leverage the strengths of both SGRT and IGRT.^1^, ^8^

### 4.3 Limitations and future directions

The clinical evaluation is currently limited to the two largest patient groups: breast and pelvic cancers. Additionally, the retrospective nature of the clinical data analysis introduces potential confounding variables, including inter‐operator variability in patient positioning technique and differences in patient characteristics between the pre‐ and post‐LUNA 3D implementation cohorts. The sample sizes of CBCT datasets per cohort, while adequate for initial clinical evaluation, are modest compared to large multi‐institutional studies.

Assessment of treatment efficiency metrics was not reported in this study as the retrospective analysis was conducted post‐clinical implementation of LUNA 3D. The lack of setup‐time data from the pre‐implementation period precludes a reliable comparison. Although not quantitatively measured in this study, radiation therapists consistently reported perceived workflow improvements and reduced setup times with LUNA 3D. In their setup time analysis, Kang et al. revealed efficiency gains with SGRT (5.2 min vs. 5.5 min),^17^ consistent with Jeong et al.’s findings of reduced positioning times (261 s vs. 281 s).^14^ It is suggested that progressive workflow improvement over time after SGRT implementation could result in further improvement of efficiency.

The version of LUNA 3D software tested during commissioning had limited interoperability with the linear accelerator control system and did not support dynamic or gated delivery. The commissioning of beam‐hold, tracking, and respiratory gating is deferred until these features are released. Once software updates enabling full linear accelerator interoperability are available, a detailed assessment of LUNA 3D's dynamic performance will be conducted and presented in a follow‐up report.

## 5 CONCLUSION

The commissioning and initial clinical evaluation of the LUNA 3D surface‐guided radiation therapy system demonstrated high accuracy, stability, and reproducibility under both phantom testing conditions and routine clinical use.

Clinical implementation of LUNA 3D resulted in site‐specific improvements in initial positioning accuracy: a significant reduction in the 3D translational vector for breast treatments and in the 3D rotational vector for pelvic treatments (both *p* < 0.001, medium effect sizes).

This study provides initial benchmark data for centers implementing the LUNA 3D system and demonstrates that modern SGRT technology can enhance positioning accuracy in routine clinical practice. The findings contribute to the growing body of evidence supporting SGRT as an increasingly used component of modern radiotherapy quality management.

## AUTHOR CONTRIBUTIONS

**Hui Khee Looe**: Conceptualization (lead); methodology (lead); project administration (equal); supervision (equal); investigation (equal); formal analysis (equal); visualization (equal); validation (equal); writing—original draft (lead). **Niklas Felix Hendrik Bartner**: Investigation (equal); formal analysis (equal); visualization (equal); validation (equal); writing—review & editing (equal). **Björn Poppe**: Conceptualization (equal); methodology (equal); project administration (equal); supervision (equal); writing—review & editing (equal). **Kay C. Willborn**: Conceptualization (equal); methodology (equal); project administration (equal); supervision (equal); writing—review & editing (equal).

## CONFLICT OF INTEREST STATEMENT

The authors declare that they have no conflicts of interest or financial interests to disclose. The LUNA 3D system evaluated in this study was independently purchased for routine clinical use.

## Data Availability

Authors will share data upon request to the corresponding author.

## References

[1] Freislederer P , Batista V , Öllers M , et al. ESTRO‐ACROP guideline on surface guided radiation therapy. Radiother Oncol. 2022;173:188‐196. doi:10.1016/j.radonc.2022.05.026 35661677 10.1016/j.radonc.2022.05.026

[2] Freislederer P , Kügele M , Öllers M , et al. Recent advanced in surface guided radiation therapy. Radiat Oncol. 2020;15(1):187. doi:10.1186/s13014‐020‐01629‐w 32736570 10.1186/s13014-020-01629-w PMC7393906

[3] Sánchez‐Rubio P , Rodríguez‐Romero R , Pinto‐Monedero M , Alejo‐Luque L , Martínez‐Ortega J . New findings on clinical experience on surface‐guided radiotherapy for frameless non‐coplanar stereotactic radiosurgery treatments. J Appl Clin Med Phys. 2024;25(12):e14510. doi:10.1002/acm2.14510 39287562 10.1002/acm2.14510 PMC11633809

[4] Naidoo W , Leech M . Feasibility of surface guided radiotherapy for patient positioning in breast radiotherapy versus conventional tattoo‐based setups—a systematic review. Tech Innov Patient Support Radiat Oncol. 2022;22:39‐49. doi:10.1016/j.tipsro.2022.03.001 35481261 10.1016/j.tipsro.2022.03.001 PMC9035716

[5] Darréon J , Massabeau C , Geffroy C , Maroun P , Simon L . Surface‐guided radiotherapy overview: technical aspects and clinical applications. Cancer Radiother. 2023;27(6‐7):504‐510. doi:10.1016/j.canrad.2023.07.003 37558608 10.1016/j.canrad.2023.07.003

[6] Bellala R , Kuppusamy A , Bellala VM , et al. Review of clinical applications and challenges with surface‐guided radiation therapy. J Cancer Res Ther. 2023;19(5):1160‐1169. doi:10.4103/jcrt.JCRT_1147_21 37787279 10.4103/jcrt.JCRT_1147_21

[7] Rudat V , Shi Y , Zhao R , Xu S , Yu W . Setup accuracy and margins for surface‐guided radiotherapy (SGRT) of head, thorax, abdomen, and pelvic target volumes. Sci Rep. 2023;13(1):17018. doi:10.1038/s41598‐023‐44320‐2 37813917 10.1038/s41598-023-44320-2 PMC10562432

[8] Al‐Hallaq HA , Cerviño L , Gutierrez AN , et al. AAPM task group report 302: surface‐guided radiotherapy. Med Phys. 2022;49(4):e82‐e112. doi:10.1002/mp.15532 35179229 10.1002/mp.15532 PMC9314008

[9] Poppinga D , Kretschmer J , Brodbek L , Meyners J , Poppe B , Looe HK . Evaluation of the RUBY modular QA phantom for planar and non‐coplanar VMAT and stereotactic radiations. J Appl Clin Med Phys. 2020;21(10):69‐79. doi:10.1002/acm2.13006 10.1002/acm2.13006 PMC7592965 32797670

[10] Brodbek L , Kretschmer J , Büsing K , Looe HK , Poppe B , Poppinga D . Systematic end‐to‐end testing of multiple target treatments using the modular RUBY phantom. Biomed Phys Eng Express. 2021;8(1):015018. doi:10.1088/2057‐1976/ac3e37 10.1088/2057-1976/ac3e37 34844222

[11] Klein EE , Hanley J , Bayouth J , et al. Task group 142 report: quality assurance of medical accelerators. Med Phys. 2009;36(9):4197‐4212. doi:10.1118/1.3190392 19810494 10.1118/1.3190392

[12] Liu X , Alexander D , Kayser D , Speck T , Wiersma RD . A novel SGRT system for real‐time optical surface tracking or guidance. J Appl Clin Med Phys. 2025;26(7):e70138. doi:10.1002/acm2.70138 40653756 10.1002/acm2.70138 PMC12256682

[13] Lew KS , Lee JKH , Chua CGA , et al. Commissioning of LUNA three‐dimensional surface‐guided radiotherapy in full rotating gantry proton therapy system. Int J Part Ther. 2026;19:101303. doi:10.1016/j.ijpt.2026.101303 41756749 10.1016/j.ijpt.2026.101303 PMC12934292

[14] Jeong Y , Kim JH , Oh JG , Lee KK , Moon SR . Comparative study on setup accuracy of marker‐less surface‐guided radiation therapy versus traditional skin marks in hypofractionated whole breast irradiation: a marker‐free approach starting from CT simulation. J Radiat Res Appl Sci. 2025;18(1):101290. doi:10.1016/j.jrras.2025.101290

[15] Svestad JG , Heydari M , Mikalsen SG , Flote VG , Nordby F , Hellebust TP . Surface‐guided positioning eliminates the need for skin markers in radiotherapy of right sided breast cancer: a single center randomized crossover trial. Radiother Oncol. 2022;177:46‐52. doi:10.1016/j.radonc.2022.10.017 36309152 10.1016/j.radonc.2022.10.017

[16] Rigley J , Robertson P , Scattergood L . Radiotherapy without tattoos: could this work. Radiography (Lond). 2020;26(4):288‐293. doi:10.1016/j.radi.2020.02.008 32245712 10.1016/j.radi.2020.02.008

[17] Kang S , Jin H , Chang JH , et al. Evaluation of initial patient setup methods for breast cancer between surface‐guided radiation therapy and laser alignment based on skin marking in the Halcyon system. Radiat Oncol. 2023;18(1):60. doi:10.1186/s13014‐023‐02250‐3 37016351 10.1186/s13014-023-02250-3 PMC10071653

